# Amputees but not healthy subjects optimally integrate non-spatially matched visuo-tactile stimuli

**DOI:** 10.1016/j.isci.2024.111685

**Published:** 2024-12-25

**Authors:** Giuseppe Valerio Aurucci, Greta Preatoni, Gaia Risso, Stanisa Raspopovic

**Affiliations:** 1 Laboratory for Neuroengineering, Department of Health Science and Technology, Institute for Robotics and Intelligent Systems, ETH Zürich, 8092 Zürich, Switzerland; 2 Institute of Health, School of Health Sciences, HES-SO Valais-Wallis, 1950 Sion, Switzerland; 3 The Sense Innovation & Research Center, 1950 Sion and Lausanne, Switzerland

**Keywords:** Clinical neuroscience, Sensory neuroscience

## Abstract

Our brain combines sensory inputs to create a univocal perception, enhanced when stimuli originate from the same location. Following amputation, distorted body representations may disrupt visuo-tactile integration at the amputated leg. We aim to unveil the principles guiding optimal and cognitive-efficient visuo-tactile integration at both intact and amputated legs. Hence, we designed a VR electro-stimulating platform to assess the functional and cognitive correlates of visuo-tactile integration in two amputees and sixteen healthy subjects performing a 2-alternative forced choice (2AFC) task. We showed that amputees optimally integrate non-spatially matched stimuli at the amputated leg but not the intact leg (tactile cue at the stump/thigh and visual cue under the virtual foot), while healthy controls only integrated spatially matched visuo-tactile stimuli. Optimal integration also reduced 2AFC task reaction times and was confirmed by cognitive EEG-based mental workload reduction. These findings offer insights into multisensory integration processes, opening new perspectives on amputees’ brain plasticity.

## Highlights

• Amputees optimally integrate non-spatially matched cues at the amputated leg only
• Instead, healthy controls optimally integrate spatially matched cues only
• Optimal multisensory integration results in reduced reaction times of the 2AFC task
• Optimal integration lowers cognitive effort, confirmed by the EEG workload index

## Graphical abstract

**Figure:**
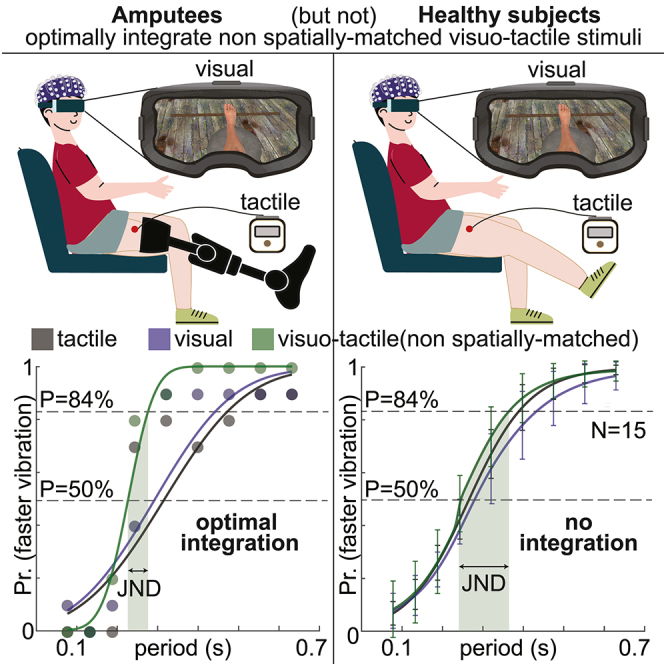

## Introduction

When we interact with the environment, we receive inputs from different sensory modalities. Our brain evaluates complementary and redundant information to generate a coherent percept in the so-called process of “multisensory integration”.^1^^,^^2^^,^^3^ Researchers have extensively examined models of multisensory integration, with Ernst and Banks'^3^ maximum-likelihood estimation theory playing a pivotal role in shaping our understanding. Recently, computational and theoretical framework based on Bayesian models has been exploited to measure and explain the key role of the multisensory integration of bodily signals for the experience of the body as one’s own, i.e., “body ownership.”^4^^,^^5^ When integrating bodily information coming from multiple sensory modalities, the final percept emerges from a weighted combination of unimodal cues (where the weights correspond to the reliability of each stimulus) which minimizes the variance of the estimation and accounts for body ownership.

When facing an amputation the brain undergoes drastic changes; the normal flow of sensory information is disrupted, and amputees commonly report feelings of ownership toward the phantom missing limb, often resulting in painful sensations.^6^ Despite multiple theories on what causes phantom experiences, there is still no consensus on the driving mechanisms underlying them.^7^ Notably, the artificial sensory feedback implemented in bionic upper and lower limbs has been proven to be effective in reducing the abnormal perception of the body.^8^^,^^9^^,^^10^^,^^11^^,^^12^ Studies suggest that this may directly impact the principles of multisensory integration, as neural stimulation enables the re-establishment of optimally integrated sensory feedback, following the same computational rules observed in the healthy nervous system.^3^^,^^4^ By presenting multisensory visuo-tactile stimuli Risso et al.^10^ showed not only that amputees can optimally integrate natural and artificial sensory information coming from the prosthesis but also that the multisensory stimulation decreases phantom limb distortions. The result was surprising, given that the integrated stimuli were spatially mismatched: the tactile stimulus was delivered on the stump of the subject, while the visual cue was provided at the foot of an embodied virtual avatar. Classical principles of multisensory integration state that stimuli are integrated across modalities only if they are presented close in space and time, i.e.,: the “binding window” is spatio-temporally constrained.^1^^,^^13^^,^^14^ This process has been well documented also at single neurons in the superior colliculus, which synthesizes concordant combinations of multisensory signals to enhance the vigor of their responses.^15^^,^^16^^,^^17^ However, this capability is not an innate feature of the circuit and requires the acquirement of postnatal experience with multisensory cues.^18^^,^^19^ Furthermore, the integrity of the spatial rule has been shown to highly depend on specific task performed.^20^^,^^21^ Indeed, the integration may be influenced by the stimuli characteristics such as crossmodal correspondence, referred to as the high-level matching of features across sensory modalities,^22^^,^^23^ and the semantic congruency, i.e., the congruent matching of unimodal stimuli with features semantically mapped to a specific object.^24^^,^^25^^,^^26^ Also, while the spatial rule is respected in the majority of spatially dependent tasks (such as a speeded orienting task to fixate target stimuli), several studies failed to demonstrate the validity of the spatial rule in non-spatially dependent tasks,^20^ as the one proposed by Risso et al..^10^ Taken together, all these findings suggest that the spatial rule, often considered as a bottom-up constraint on multisensory integration in humans, might instead represent a top-down operation. In other words, it would consist of a cognitive factor (i.e., a prior, according to the Bayesian framework) informing the observer that the multisensory cues belong together.

With reference to the case of amputees, the spatial flexibility in the integration of sensory stimuli observed in ^10^ could be a reflection of two results. On one hand, the fact that the developed multimodal platform is able to strongly modulate the cognitive experience of all subjects, creates the powerful illusion that the virtual object and the body are a single entity. On the other hand, the flexibility observed in the two individuals could be a peculiar characteristic of amputees. In other words, the increased flexibility in the spatial integration of multisensory stimuli might reflect neural plasticity mechanisms triggered by the limb loss. The absence of any control condition in ^10^, prevented far-reaching conclusions regarding the direct connection between the amputation and the disruption of the spatial rule. Here, to understand how the limb loss may affect brain processing strategies of sensory signals, we test the multisensory integration of i) two amputees, both on the stump and on their intact legs, ii) 16 healthy individuals, presenting stimuli that were spatially matched and non-spatially matched.

To quantitatively investigate multisensory integration, we here propose a rigorous psychophysical approach relying on a two-forced alternative (2AFC) task repeated over unimodal and bimodal conditions.^10^^,^^27^ Alongside, to strengthen the experimental results, participants' reaction times were recorded, and EEG correlates of multisensory integration were collected in all participants.

Several studies have tried to establish a link between multisensory integration and subjects’ EEG-based cognitive workload,^28^^,^^29^ defined as the amount of cognitive resources to be employed during the completion of a task.^30^^,^^31^^,^^32^ Marucci et al.^28^ demonstrated the decrease of an EEG-based mental workload index indicating the facilitatory impact of multimodal stimuli over unimodal cues during the competition of Virtual-reality (VR) based tasks. This multimodal evaluation of multisensory integration is crucial to deepening our knowledge of its intricate processes, extending from the mere perception of external stimuli to the core of our bodily self-consciousness and representation.

Taken together, the proposed study aims to provide robust behavioral, functional, and neurophysiological indicators of the mechanisms governing multisensory integration strategies in amputees, offering functional evidence of brain plasticity changes resulting from limb loss.

## Results

### Optimal integration of non-spatially matched visuo-tactile stimuli occurs at the amputated leg, but not at intact legs in either amputees or healthy controls

Two transfemoral male amputees (29 and 58 years old) and sixteen healthy subjects (8 females and 8 males, mean age of 27±10) participated in the study. To assess optimal visuo-tactile stimulus integration, we applied the statistical approach proposed by Ernst and Banks in 2002.^3^ According to the authors, in a multisensory task, humans assign the sensory reliabilities of unisensory cues to construct an optimal behavior, representing the lowest-variance estimate of the bimodal value. Therefore, optimal integration is verified when the observed value does not differ from the predicted optimal value. To test for the optimal integration of spatially and non-spatially matched stimuli in amputees and healthy subjects, the experimental design was modified according to our hypotheses.

**Figure 1:**
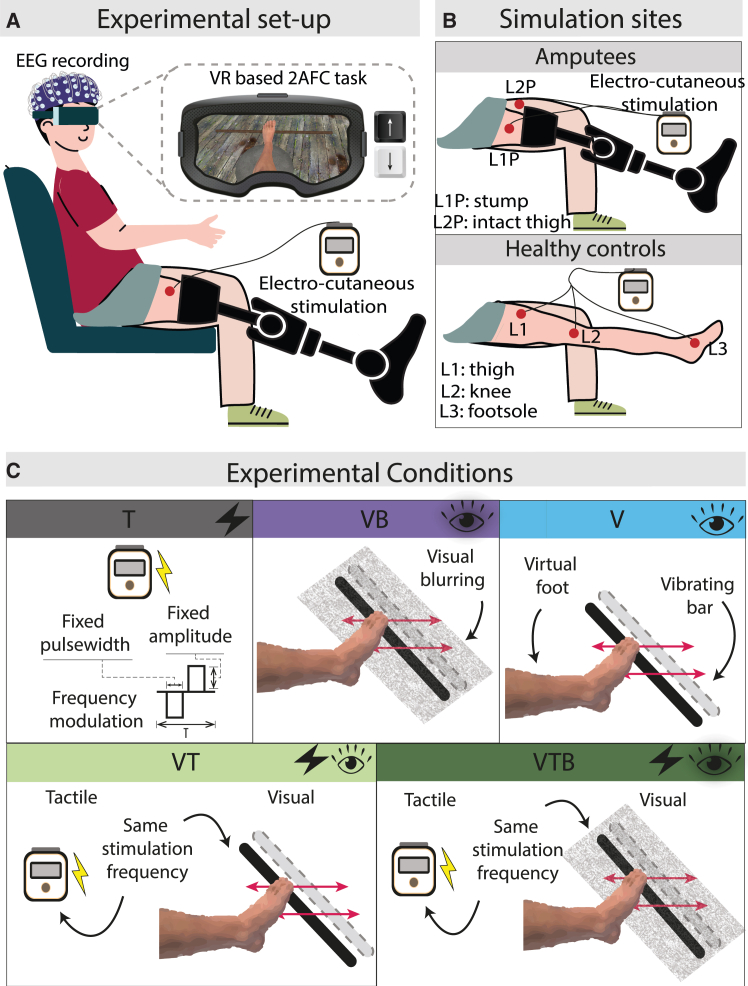
Experimental set-up and experimental conditions (A) Experimental set-up. Subjects wore the EEG cap and were immersed in the VR scene showing themselves from a first-person perspective while performing a 2-AFC Task. The task combined visual (VR) and tactile (Electro-cutaneous stimulation) cues. (B) Stimulation sites. For patients, electrical stimulation was delivered at the frontal part of the stump (Location1Patients L1P) and at the frontal part of the thigh of the intact leg (Location2Patients L2P). For healthy controls, the stimulation sites were the upper thigh (L1), the knee (L2), and the foot sole (L3). (C) Experimental conditions. Subjects performed the 2-AFC task under five experimental conditions: Tactile (T), Visual (V), Visual Blurred (VB), Visuo-tactile (VT), and Visuo-Tactile Blurred (VTB).

Participants were fully immersed in a first-person perspective VR scenario and performed a two-alternative forced-choice (2AFC) task, judging which of two sequential stimuli had the higher frequency. Each stimulus lasted 1.5 s, with a 0.5 s interval between them. After the second stimulus concluded, participants indicated their choice on a computer keyboard, selecting the upward arrow if they perceived the first stimulus as faster or the downward arrow for the second. Stimuli types included unimodal (either tactile or visual) and bimodal (visuo-tactile) presentations. Visual stimuli consisted of the vibration of a wooden bar placed below the virtual avatar’s foot, and tactile cues were electrical stimulations provided in different locations (Figures 1A and 1B). Given the predominance of vision over the tactile input, a visual blur condition was introduced using a blurred panel to avoid the winner-take-all hypothesis,^10^^,^^33^ where one sensory cue prevails over the other. As a result, five conditions were tested: Visual (V), Tactile (T), Visuo-Tactile (VT), Visual Blurred (VB), and Visual Blurred-Tactile (VTB) (Figure 1C).

In amputees, to test spatial constraints on multisensory integration, the conditions were repeated by providing the electrical stimulation in two anatomical sites: the frontal part of the stump (L1P, non-spatially matched) and the thigh of the non-amputated leg (L2P, non-spatially matched) (Figure 1B). For healthy subjects’ testing, three sites were chosen: frontal thigh (L1, non-spatially matched with the visual stimulus), knee (L2, non-spatially matched), and footsole (L3, spatially matched) (Figure 1B). In all conditions, the visual stimulus location was always the same (i.e., vibration of a wooden bar placed below the virtual avatar’s foot).

For each condition and position, a psychometric curve was fitted using a probit regression. This curve illustrates the probability of perceiving the comparison stimulus as having a longer vibrating period than a reference stimulus. The Point of Subjective Equality (PSE) was extracted as the vibrating period at which the comparison stimulus is perceived as longer than the reference period in 50% of trials. The Just Noticeable Difference (JND) was calculated as the difference between the PSE and the vibrating period perceived as longer than the reference in 84% of trials.^10^

**Figure 2:**
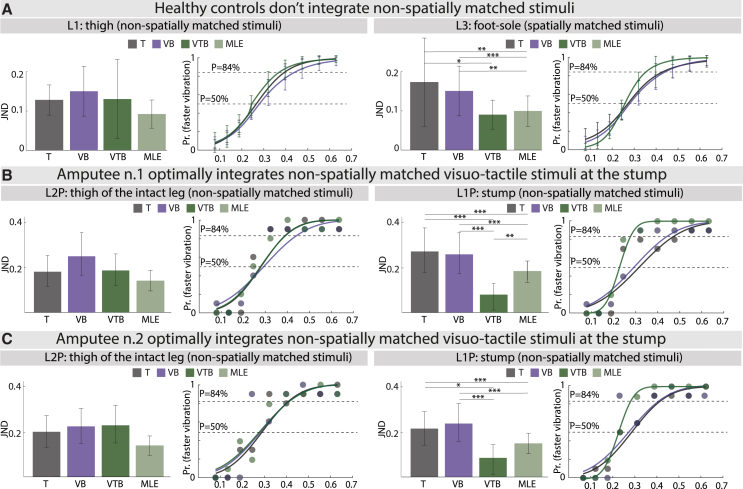
Statistical results of the multisensory integration (A) Healthy subjects’ JND results. Healthy subjects’ JND results and psychometric curves for the blurred conditions (T, VB, VTB) across L1 (left) and L3 (right) stimulation sites. For JND, the predicted maximum likelihood estimation (MLE) JND is shown as well. Repeated measures ANOVA tests with “lsd” post hoc were used (*N* = 15). Mean +/− STD is shown for each bar. The psychometric curves shown (T, VB, VTB) correspond to the average psychometric curves across *N* = 15 subjects. The 50% and 84% probability thresholds are shown as horizontal lines. (B and C) Amputee n.1 (B) and Amputee n.2 (C) JND results. Amputees’ JND and psychometric curves for blurred conditions (T, VB, VTB) across the L1P (right) and L2P (left) stimulation sites. For JND, the predicted MLE JND is shown as well. All bar plots correspond to the bootstrapped distributions (*N* = 5000). For each bar, the mean and 95% confidence intervals are shown. False Discovery Rate adjusted bootstrap *p*-value are reported. In the psychometric curves, the 50% and 84% probability thresholds are shown as horizontal lines. For all plots: ∗*p* < 0.05, ∗∗*p* < 0.01, ∗∗∗*p* < 0.001.

In healthy subjects, as expected, the visual JND was statistically lower than the tactile JND in all the positions (Tables S1 and S2). Optimal multisensory integration was then assessed on the blurred condition. Only for the spatially matched site L3 (foot-sole), results pointed at the optimal integration of the unimodal cues (${JND}_{T,L3}^{healthy}=0.173\pm 0.113,{JND}_{VB,L3}^{healthy}=0.15\pm 0.063,{JND}_{VTB,L3}^{healthy}=0.09\pm 0.037,{JND}_{MLE,L3}^{healthy}=0.099\pm 0.04,ANOVA\;F=5.85,ANOVA\;p_{value}=0.002,p_{value,T-VTB}=0.026,p_{value,VB-VTB}=0.004,p_{value,VTB-VTBP}=0.57$) (Figure 2A, Tables S1 and S2). In the other sites, the bimodal JND was never statistically different from both the unimodal JNDs (Figures 2A and S1, Tables S1 and S2).

For the first amputee, the visual JND was statistically lower than the tactile JND in both positions (Tables S1 and S3). As expected, the visual cue was significantly more reliable than the tactile cue and the winner-take-all hypothesis prevented any further analysis and interpretation. For the blurred condition, at the level of the stump (L1P), the observed bimodal condition ${\;JND}_{VTB,L1P}^{patient1}=0.078\;\left[0.009,0.120\right]$ was statistically different from both the unimodal cues (${JND}_{T,L1P}^{patient1}=0.264\;\left[0.173,0.372\right],{JND}_{VB,L1P}^{patient1}=0.252\;\left[0.169,0.352\right],p_{values}<0.001$) and lower than the predicted value $(\;{JND}_{MLE,L1P}^{patient1}=0.178\;\left[0.133,0.228\right]$), indicating the optimal integration of the unimodal stimuli (Figure 2B, Tables S1 and S3). For the intact leg instead, unimodal JNDs (${JND}_{T,L2P}^{patient1}=0.183\;\left[0.117,0.256\right],{JND}_{VB,L2P}^{patient1}=0.253\;\left[0.167,0.359\right]$ were not statically different from the observed bimodal JND (${JND}_{VTB,L2P}^{patient1}=0.189\;\left[0.12,0.263\right],p_{values}>0.05)$ (Figures 2B and S2, Tables S1 and S3).

Similar results were observed for the second amputee. In the nonblurred conditions, the visual cue was significantly more reliable than the tactile one at the stump (Tables S1 and S4). Multisensory integration was then assessed in the blurred conditions. Again, optimal integration was achieved at the amputated leg: the observed bimodal JND condition ${\;JND}_{VTB,L1P}^{patient2}=0.083\;\left[0.010,0.128\right]$ was statistically different from both the unimodal cues (${JND}_{T,L1P}^{patient2}=0.210\;\left[0.138,0.289\right],{JND}_{VB,L1P}^{patient2}=0.232\;\left[0.153,0.320\right],p_{values}<0.05)$ but not statistically different from the predicted value $(\;{JND}_{MLE,L1P}^{patient2}=0.152\;\left[0.112,0.194\right]$, $p_{value}=0.067)$. For the intact leg, unimodal JNDs (${JND}_{T,L2P}^{patient2}=0.207\;\left[0.136,0.287\right],{JND}_{VB,L2P}^{patient2}=0.232\;\left[0.153,0.320\right]\;)$ were not statically different from the observed bimodal JND (${JND}_{VTB,L2P}^{patient2}=0.237\;\left[0.156,0.334\right],p_{values}>0.05)$ (Figures 2C and S2, Tables S1 and S4).

### Functional and cognitive outcomes of multisensory integration

**Figure 3:**
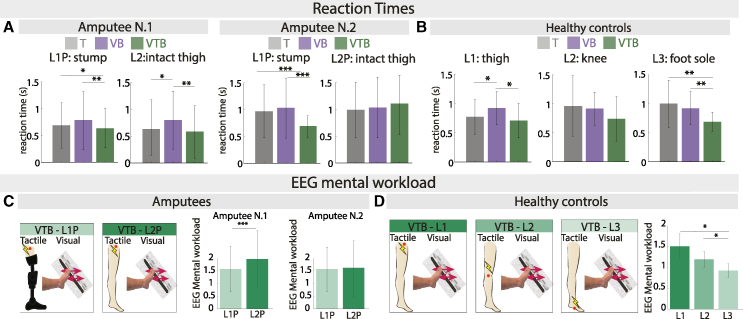
Functional and cognitive outcomes of multisensory integration (A) Amputees’ reaction times results. Amputees’ reaction times during the 2-AFC tasks for both stimulation sites (L1P, L2P) for unimodal (T, VB) and bimodal (VTB) conditions are reported. Mean +/− STD is shown for each bar. Friedman tests with “lsd” post hoc was used (*N* = 80 repetitions). (B) Healthy subjects' reaction time results. Reaction times for the three stimulation sites (L1, L2, L3) are shown. Mean +/− STD is shown for each bar. Repeated measures ANOVA tests with “lsd” post hoc were used (*N* = 16 subjects). (C) Amputees’ EEG mental workload. EEG mental workload for the bimodal VTB condition are reported. Mean +/− STD are shown for each bar. Friedman tests (*N* = 176) with “lsd” post hoc were used. (D) Healthy subjects’ EEG mental workload. EEG mental workload for the bimodal VTB condition is reported. Mean +/− STD is shown for each bar. Repeated measures ANOVA (*N* = 14) tests with “lsd” post hoc were used. For all plots: ∗*p* < 0.05, ∗∗*p* < 0.01, ∗∗∗*p* < 0.001.

To assess the functional outcomes of the multisensory integration, reaction times were extracted. They were computed as the interval between the termination of the second stimulus and the instant at which the participant provided their response, for each stimulation site and experimental condition. For both amputees, the reaction times for the amputated leg (L1P) were significantly lower in the bimodal condition (${RT}_{VTB,L1P}^{patient1}=0.64\pm 0.37\;s\;\left(mean\;\pm std\right),{RT}_{VTB,L1P}^{patient2}=0.69\pm 0.20\;s)$ compared to the unimodal conditions (${RT}_{T,L1P}^{patient1}=0.69\pm 0.43\;s,{RT}_{VB,L1P}^{patient1}=0.79\pm 0.55\;s,Friedman\;\chi^{2}=9.38,{Friedman\;p}_{value}=0.009,p_{value,T-VTB}=0.036,p_{value,VB-VTB}=0.003\;N=77repetitions;\;{RT}_{T,L1P}^{patient2}=0.97\pm 0.51\;s,{RT}_{VB,L1P}^{patient2}=1.03\pm 0.57\;s,Friedman\;\chi^{2}=9.12,{Friedman\;p}_{value}=0.011,p_{value,T-VTB}<\;0.001,p_{value,VB-VTB}<\;0.001,N=77\;repetitions)$ (Figure 3A, Tables S5 and S6). For both amputees, this did not happen in the case of the intact leg (L2P) where the reaction time of at least one of the unimodal conditions was not statistically higher than the reaction time of the bimodal condition (${RT}_{T,L2P}^{patient1}=0.62\pm 0.48\;s,{RT}_{VB,P2P}^{patient1}=0.79\pm 0.55\;s,{RT}_{VTB,L2P}^{patient1}=0.59\pm 0.51\;s,Friedman\;\chi^{2}=26.8,{Friedman\;p}_{value}<0.001,p_{value,T-VTB}=0.468,p_{value,VB-VTB}=0.004,N=77\;repetitions;\;{RT}_{T,L2P}^{patient2}=0.99\pm 0.51\;s,{RT}_{VB,L2P}^{patient2}=1.03\pm 0.57\;s,{RT}_{VTB,L2P}^{patient2}=1.10\pm 0.56\;s,Friedman\;\chi^{2}=0.96,{Friedman\;p}_{value}=0.62,N=77\;repetitions$) (Figure 3A, Tables S5 and S6). For the healthy control subjects, the reaction time in the bimodal VTB condition showed a significant decrease compared to the unimodal conditions when the sensory cues were spatially matched $({RT}_{T,L3}^{healthy}=1.11\pm 0.49\;s,{RT}_{VB,L3}^{healthy}=1.03\pm 0.36\;s,{RT}_{VTB,L3}^{healthy}=0.72\pm 0.21\;s,{ANOVA\;F=6.887,ANOVA\;p=0.003,p}_{value,T-VTB}=0.003,p_{value,VB-VTB}=0.004,N=16\;subjects$) (Figure 3B, Table S7). When the multisensory cues were not spatially aligned, they did not reflect a functional improvement in reaction times. This was visible for the knee condition (L2) $({RT}_{T,P2}^{healthy}=1.04\pm 0.66\;s,{RT}_{VB,L2}^{healthy}=1.03\pm 0.36\;s,{RT}_{VTB,L2}^{healthy}=0.80\pm 0.46\;s,ANOVA\;F=2.63,ANOVA\;p=0.088,N=16\;subjects$) (Figure 3B) and for the thigh condition (L1) (${RT}_{T,L1}^{healthy}=0.83\pm 0.34,{RT}_{VB,L1}^{healthy}=1.03\pm 0.36,{RT}_{VTB,L1}^{healthy}=0.73\pm 0.31,ANOVA\;F=5.89,ANOVA\;p=0.007\;p_{value,T-VTB}=0.159)$ (Figure 3B, Table S7). All reaction time (RT) results obtained for both amputees and healthy controls were further supported by an additional Race Model (RM) analysis, which could be found in supplemental information (Figure S3).

To unveil the cognitive correlates of multisensory integration, we derived the EEG-based mental workload index by calculating the ratio between theta power in the frontal area and alpha power in the parietal area (See STAR Methods). The mental workload corresponding to the bimodal VTB was compared across the two stimulation sites. The first amputee showed a decreased mental workload in L1P, compared to L2P, hence suggesting a decreased cognitive load in processing the non-spatially matched stimuli at the level of the amputated leg (${EEGworkload}_{VTB.L1P}^{patient1}=1.58\pm 0.92,{EEGworkload}_{VTB.L1P}^{patient1}=1.98\pm 1.08,p_{value}<\;0.001,Cohen\;d=0.19,N=176)$ (Figure 3C). For the second amputee instead, no statistical differences were observed across the two stimulation sites (${EEGworkload}_{VTB.L1P}^{patient2}=1.56\pm 0.85,{EEGworkload}_{VTB,L2P}^{patient2}=1.56\pm 1.12,p_{value}=0.445,N=176)$ (Figure 3C). In healthy subjects, a reduction in mental workload was observed as the spatial proximity of unisensory cues increased $({EEGworkload}_{VTB.L1}^{healthy}=1.50\pm 0.30$, ${EEGworkload}_{VTB.L2}^{healthy}=1.18\pm 0.18,{EEGworkload}_{VTB.L3}^{healthy}=0.92\pm 0.16$) (Figure 3D). The mental workload in L3 was significantly lower compared to L1 and L2 ($ANOVA\;F=4.63,{\;ANOVA\;p}_{value}=0.019,p_{value,L1-L3}=0.021,p_{value,L2-L3}=0.026,N=14$) (Figure 3D, Table S8).

## Discussion

In this study, we aimed to investigate computational changes in the integration of multisensory stimuli after limb loss. The approach to assessing multisensory integration was multimodal: we compared observed behavior to maximum-likelihood estimated behavior (Ernst and Banks, 2002), analyzed reaction times of unimodal and bimodal cues, and extracted EEG-based markers of mental workload as neural correlates of multisensory integration.

Our key finding reveals that amputees integrate non-spatially matched visuo-tactile stimuli at the level of the amputated leg in a statistically optimal fashion, while they do not integrate the same stimuli at the level of the healthy leg (electrical stimulation at the stump/thigh and visual stimulation under the virtual foot). For healthy subjects, statically optimal integration was achieved only with spatially matched stimuli (electrical stimulation and visual stimulation under the foot). For amputees, the obtained data were strengthened by the reaction time evaluation, which pointed in the same direction as the psychophysical results, highlighting functional multisensory facilitation at the level of the stump for amputees, but not for the healthy leg. A similar enhancement was shown for healthy subjects in position L3, but not in L1 and L2, where the unisensory cues were not spatially matched.

As a primary consideration, we exclude that the obtained results are influenced by the nature of the task.^20^ Chen and Spence,^21^ identified three key perceptual mechanisms through which the human brain combines multisensory cues: 1) the unity assumption, i.e., the assumption that stimuli originate from the same spatiotemporal event^14^^,^^34^; 2) the crossmodal correspondence^22^^,^^23^; and 3) the semantic congruency.^24^^,^^25^^,^^26^ Given that crossmodal correspondence and semantic congruency depend on the type and nature of the multisensory stimulus, if they were the factors driving the integration of non-spatially matched stimuli, their influence would be evident in the 16 healthy controls or the healthy leg of amputees. Thus, we hypothesize that the primary factor contributing to optimal integration in our setup is the “unity assumption,” rather than any correlations among the unisensory stimuli received. Since our body, of which we have prior experience and knowledge, is the object of multisensory integration, the discussion of the unity assumption needs a deeper investigation of the Bayesian probabilistic approaches to body ownership.^4^^,^^5^^,^^35^ According to this approach, the feeling of owning a body, i.e., body ownership, emerges from the integration of multisensory bodily stimuli (weighted according to specific rules) that are interpreted by the brain as originating from the same common physical cause.^36^ In other words, before processing multisensory stimuli, the brain infers the likelihood that they originate from the same cause, based on their spatial and temporal congruencies.^37^ The results of this study show clear differences in the computational processes related to the amputated and intact leg (in both amputees and healthy subjects). The healthy nervous system interprets spatially non-congruent sensory signals as not coming from the same object of perception. In contrast, at the amputated limb, there is greater flexibility and the sensory signals are integrated regardless of their spatial discrepancy. This suggests that following amputation, the nervous system undergoes plastic changes that affect the interpretation and processing strategies of sensory signals from the environment.

These results provide functional evidence contributing to current discussions on cortical reorganization. Over the years, the most accredited cause for these altered experiences of the body has commonly been attributed to maladaptive plasticity.^7^ Maladaptive plasticity theory specifically refers to the fact that following the sensory deprivation after amputation, the deprived hand area of the primary sensorimotor cortex becomes responsive to inputs from cortical neighbors.^38^^,^^39^ However, recent evidence highlights the persistent representation of the missing hand,^40^^,^^41^^,^^42^ and the classical view on cortical reorganization begins to be questioned.^7^^,^^43^^,^^44^ Our results also might suggest that evidence of plasticity should be sought rather than in the cortical “hardware,” i.e., physical changes in brain matter, in the cortical “software,” i.e., in the way the brain processes and computes information. This is consistent with the hypothesis posed in Makin & Krakauer 2023^43^ according to which brain plasticity is better interpreted as the upregulation of a more general input-agnostic computational capacity that then favors one input over another. In the case of processing body-related stimuli, this redirection of computational capacities might have a direct effect on body consciousness and representation. Noteworthy, these results are not only relevant from a theoretical perspective, but they have direct implications for rehabilitation. In fact, neurorehabilitation systems that exploited neurostimulation to re-establish a regular multisensory flow of information have shown beneficial effects regarding the possibility of restoring altered body experience.^8^^,^^9^^,^^11^^,^^45^^,^^46^^,^^47^^,^^48^^,^^49^ Our findings of the successful integration of delivered neurostimulation may further explain, for instance, the results of Risso et al.,^10^ who demonstrated that synchronous visuotactile stimulation at the stump effectively reduces perceptual distortion, and the results of Chee et al.,^45^ who showed that electrical stimulation at the stump, used as sensory feedback for prosthetic foot-ground contact, led to functional walking benefits and metabolic improvement.

The proposed integration results were further supported by neurophysiological correlates. Examining the EEG correlates associated with integration indeed, the first amputee exhibited diminished mental workload in the bimodal condition for the amputated leg compared to the non-amputated leg. Despite both sites being mismatched with the visual cue, such a decrease implies that lower mental resources are needed in the former. Once again, this reduction could derive from the subjects' presumption that both sensory modalities originated from a common source. Notably, the validity of such a measure was confirmed by the results of healthy subjects, which showed reduced mental workload as the multisensory cues became closer to each other. In simple terms, we could hypothesize that when the cues were closer together, the task became both easier and more immersive.^28^

### Limitations of the study

The study presents several limitations. Due to time limitations, we were unable to replicate all tested positions for healthy subjects (L1 thigh, L2 knee, L3 footsole) in the amputees' group. Despite recognizing that including these additional positions would have double-proven the results obtained from healthy subjects, we maintain confidence in the consistency of the data from the 16 healthy subjects.

Furthermore, we used only one visual (V) and visually blurred (VB) conditions across all stimulation sites, effectively preventing the repetition of the condition and significantly reducing the experiment duration. Here it is worth noticing that the V and VB conditions are the same among the different stimulation sites (visual stimulus under the foot and no electrical stimulation), hence we presumed that comparable results would be attained with repeated iterations of the same task.

Finally, contrary to,^10^ we did not assess perceived limb distortion in our subjects. Measuring distortion could have provided a proprioceptive metric to correlate with the degree of multisensory integration achieved, as varying levels of distortion may affect spatial mismatch differently across individuals. Future studies should aim to combine functional measures with proprioceptive assessments of body distortion to address this gap.

## Resource availability

### Lead contact

Further information and requests for resources should be directed to the Lead Contact, Stanisa Raspopovic (nesta.fale@gmail.com).

### Materials availability

This study did not generate new unique reagents.

### Data and code availability

• All data reported in this article will be shared by the lead contact upon request.
• This article does not report the original code.
• Any additional information required to reanalyze the data reported in this article is available from the lead contact upon request.

## Acknowledgments

The authors express deep gratitude to the two amputees and sixteen healthy controls whose voluntary participation significantly contributed to advancing our research. The authors would like to express their gratitude to Dr. Giacomo Valle for his valuable insights and discussions during the early stages of this project. His contributions were instrumental in shaping the direction of our research. This project has received funding from the 10.13039/501100001711Swiss National Science Foundation (MOVEIT n.197271) and from 10.13039/501100012652ETH Zürich Foundation and the Swiss paraplegic foundation (RESC- pAInSense).

## Author contributions

G.V.A designed the study, performed the experiment, analyzed the data, performed the statistical analysis, made the figures, and wrote the article. G.P. designed the study, performed the initial experiments, and reviewed the article. G.R. designed the study and reviewed the article. S.R. designed the study, reviewed the article, supervised experiments, and managed the regulatory path.

## Declaration of interests

The authors declare no competing interests.

## STAR★Methods

### Key resources table

**Table:** 

REAGENT or RESOURCE	SOURCE	IDENTIFIER
**Software and algorithms**		

MATLAB R2024a	MathWorks	https://www.mathworks.com
Unity	Unity Technologies	https://unity.com

**Other**		

Rehamove3	Hasomed GmbH	https://hasomed.de
Circle Electrodes Pads (25 mm)	Tenscare	https://tenscare.co.uk
HTC VIVE	VIVE	https://vive.com

### Experimental model and study participant details

#### Subjects

Two transfemoral male amputees (29 and 58 years old) were recruited for the study. The limited sample size aligns with prior research on multisensory integration and cognitive facilitation in amputees^8^^,^^10^^,^^27^ where inter-subject analyses were typically conducted. Both amputees faced a traumatic amputation as a result of accidents occurring in 2018 and 2013 respectively and use a prosthetic leg in their daily life. Additional sixteen healthy controls (8 females and 8 males, mean age of 27±10) were recruited for the study. Both amputees faced a traumatic amputation as a result of accidents occurring in 2018 and 2013 respectively and use a prosthetic leg in their daily life. All participants signed a consent form. The study was conducted in line with the Helsinki Declaration of 1975, as revised in 2000 (World Medical Association Declaration of Helsinki 2000) and was approved by the ETH Ethical Commission (EK-2019-N-97).

### Method details

#### Multisensory platform

The employed multisensory platform combined Cutaneous Electrical Stimulation, provided via the RehaMove3 (Hasomed GmbH) stimulator, with the HTC VIVE Pro Virtual Reality headset. To measure neurophysiological correlates of the multisensory integration, A 24-channel portable EEG device (SMARTING MOBI, mBrain Train) with a sampling frequency of 500 Hz was employed (Figure 1A).

In the VR scenario, developed in Unity 3D, subjects saw themselves from a first-person perspective sitting on a chair. One virtual leg was slightly raised and placed on a stool. Behind the corresponding foot, a small wooden stick was placed (Figure 1A)^10^ to provide an unimodal visual stimulus. The tactile stimulus was elicited by electric biphasic charge-constant pulses with variable frequency. Two superficial electrodes of 1 cm^2^ were employed to target different anatomical positions (Figures 1A and 1B) (See STAR Methods section: experimental protocol). The connection between the VR scenario and the stimulator was ensured with a custom-script Pipe-Server built on a dynamic-link library (DLL) that was imported into the Unity C# script. At the same time, a Lab Streaming Layer (LSL) protocol allowed the precise synchronization of the different stimuli with the EEG signal.

#### 2-AFC task and experimental conditions

During the experiment, subjects were immersed in the VR scenario and performed a two-alternative forced (2AFC) task, where they had to judge which of two consecutive stimuli had the highest frequency. In the task, each of the stimuli was presented for 1.5 seconds and the pause between two consecutive stimuli was 0.5 seconds. After the end of the second stimulus, subjects could prompt on the computer keyboard which stimulus was perceived as faster (upward arrow = first stimulus, downward arrow = second stimulus). The stimulus could be either unimodal (tactile or visual) or bimodal (visuo-tactile). The visual stimulus consisted of the vibration of the wooden bar at specific frequencies (See STAR Methods section: stimuli selection in the 2-AFC task), while the tactile stimulus was achieved via biphasic charge-constant electrical stimulation at fixed frequencies (See STAR Methods section: stimuli selection in the 2-AFC task). In the unimodal conditions, subjects received either one of the two inputs. To ensure consistency among conditions, the unimodal tactile condition was still performed in the VR environment, but a black panel was placed in front of the wooden bar to prevent subjects from seeing its movement. In the bimodal visuo-tactile condition, the vibrating frequencies of the visual and tactile inputs were matched to provide consistent and congruent information. Recognizing the predominant reliance on vision over touch in humans, and to counter the tendency for subjects to overly depend on visual input, we introduced a visual blur condition.^11^^,^^50^ To achieve that, we positioned a blurred panel in front of the virtual foot of the subject, to increase the difficulty of the task (Figure 1C). This manipulation ensured equal weighting between visual and tactile inputs and allowed a meaningful use of the optimal integration model proposed by Ernst and Banks (2002). As a result, a total of five different experimental conditions were employed: Visual (V), Tactile (T), Visuo-Tactile (VT), Visual Blurred (VB), and Visual Blurred-Tactile (VTB) (Figure 1C). To test the constraint that the spatial rule poses on the multisensory integration, these experimental conditions were repeated by administering the electrical stimulation across different anatomical sites, while keeping the visual stimulus unaltered (bar vibrating under the foot). For amputees testing, electrical stimulation was provided at two sites: the frontal part of the stump (L1P Location1Patients) (as in Risso et al.), and the thigh of the non-amputated leg (L2P Location2Patients) (Figure 1B). For healthy subjects, electrical stimulation was provided at the upper-frontal thigh (L1, used in Risso et al.), at the knee (L2), and at the foot sole (L3) of the left leg (Figure 1B). Unlike the first two sites, the foot sole was spatially matched with the visual stimulus induced by the vibration of the wooden bar. The three sites were chosen to study the impact of spatial distance on multisensory integration strategies, specifically examining the effects of increasing and decreasing stimuli spatial proximity.

#### Stimuli selection in the 2-AFC task

In this study, the selection of stimulation frequencies deviates slightly from the methodology previously proposed by ^27^. To accommodate the exploration of a broader range of experimental conditions while maintaining experiment duration within reasonable limits, we reduced the number of possible frequencies from eleven to nine. The reference stimulation period, always presented as one of the two stimuli in each 2-AFC task, was fixed at 0.24442 seconds. For constructing psychometric curves, three lower periods with a step down equal to 0.05555, and five higher periods with a step up equal to 0.07777 were used as comparison stimuli. During the task, each of the nine possible stimuli combinations was randomly repeated ten times for a total of ninety pairs of stimuli.

#### Experimental protocol

All the experiments were conducted in the Laboratory. Firstly, the EEG cap was mounted on the head of the subject. High-chloride abrasive electrolyte gel was placed in each of the 24 electrodes to ensure acceptable impedance between the electrode and the scalp (< 10 KOhm). Then, the calibration of the electrical stimulation for each site of interest (L1P, L2P for amputees, and L1, L2, L3 sites for healthy subjects) was performed. During the calibration, subjects underwent three ramps of 1.5 seconds-long biphasic stimuli with fixed stimulation period (0.2442 seconds) and increasing amplitude. They were asked to report a sensation of 6 out of 10,^45^ in correspondence with which they could unambiguously perceive the administered electrical stimulation. The amplitude values were then stored for the experiments. Once the calibration was concluded, the subjects entered the VR scenario and repeated the 2-AFC task for each of the chosen electrical stimulation sites (three for healthy subjects and two for amputees) and each of the experimental conditions (V, T, VT, VB, VTB). The order of the stimulation sites, as well as the order of the experimental conditions for each stimulation site, was randomized. The precise order of conditions for each amputee and healthy controls is reported in Tables S9 and S10, respectively.

#### EEG mental workload

Both amputees and healthy subjects wore the EEG cap throughout the entire experiment duration. However, data from two healthy subjects were excluded due to connectivity issues. Initially, the EEG signal underwent visual inspection, and segments with corrupted signals were removed. Subsequently, the EEG data underwent band-pass filtering (0.5 – 42 Hz) using a Finite Impulse Response (FIR) windowed-sinc (Hamming) filter with an order of 8000. The data were then re-referenced to the common average. To eliminate artifacts arising from eye and muscle movements, Independent Component Analysis (ICA) was applied, and any corrupted components were subsequently removed. Following artifact removal, epochs of 1.75 seconds, commencing from the onset of each stimulus, were extracted. We applied this epoching process from the first stimulus of the initial condition to the last stimulus of the final condition. These epochs were then labelled based on the corresponding stimulation site and experimental conditions from which they were derived. Electrodes from frontal and parietal areas, known as key areas in cognitive load^28^^,^^30^^,^^51^ were then selected (Fz,F7,F8,Pz,P3,P4). Power Spectral Densities (PSD) were estimated using the Welch method, employing a window size of 875 ms, 50% overlap, and zero padding of 1024 samples. Individual Alpha Frequency (IAF) for each subject was determined as the frequency corresponding to the highest PSD peak within the range of 7.5 to 12.5 Hz.^51^^,^^52^ Based on the extracted IAF values, the Theta and Alpha bands were defined for each subject as follows:

• Theta Band: (IAF – 6) to (IAF – 2) Hz
• Alpha Band: (IAF – 2) to (IAF + 2) Hz

Subsequently, the mental workload metric, defined as the ratio between frontal activity in the theta band and parietal activity in the alpha band, was calculated.

$$EEG\;workload=\frac{Theta\left(Fz,F7,F8\right)}{Alpha\left(Pz,P3,P4\right)}$$

The workload metric for each experimental condition and simulating site was then calculated as the average EEG workload of all the corresponding epochs.

### Quantification and statistical analysis

For every statistical test, parametric (Repeated Measures ANOVA) or non-parametric (Friedman, Wilcoxon signed-rank) tests were used depending on the normality of the data, which was assessed with the Kolmogorov-Smirnov test. For all the plots in the Figures, the asterisk are defined as follows: ∗ p < 0.05, ∗∗ p < 0.01, ∗∗∗ p < 0.001.

#### Optimal integration model

The Maximum Likelihood Estimation (MLE) model, originally proposed by Ernst and Banks in 2002^3^ was used to assess optimal integration in multisensory perception. The model assumes that the brain combines redundant sensory information in a statistically optimal manner,^3^^,^^37^^,^^53^ whereby the final multisensory estimate $s_{vt}$ corresponds to a weighted average of the visual $s_{v}$ and tactile $s_{t}$ cues:

(Equation 1) $$s_{vt}=w_{v}s_{v}+w_{t}s_{t}$$

where the weights sum up to 1 ($w_{v}+w_{t}=1)$ and are proportional to the reliability of each specific cue:

(Equation 2) $$w_{v}=\frac{R_{v}}{R_{v}+R_{t}}\;w_{t}=\frac{R_{t}}{R_{v}+R_{t}}$$

Here, the reliability is defined as the inverse of its noise (i.e., variance) $R_{i}=\frac{1}{\sigma_{i}^{2}}$. Assuming that unisensory cues are normally distributed and independent, the MLE model predicts an optimal multisensory estimate with a variance lower than that of either single as:

(Equation 3) $$\sigma_{vt}^{2}=\frac{\sigma_{v}^{2}\;\sigma_{h}^{2}}{\sigma_{v}^{2}+\sigma_{t}^{2}}$$

In behavioral experiments, the Just-Noticeable Difference (JND), is used as a measure of sensory noise for each cue. Hence, we used the JND of the unimodal cues to predict the optimal JND of the combined cues, calculated as in Equation 3. It is worth noticing that the benefit of multisensory integration is maximized when sensory modalities have similar reliabilities.^54^ When one cue is significantly more reliable than the other, integration offers minimal improvement, and performance aligns with the more reliable cue, as predicted by the "winner-take-all" hypothesis. To counteract vision’s predominance over tactile input and prevent a winner-take-all effect, we then introduced a blurred visual condition.

#### Statistical analysis: Optimal integration

The whole analysis was conducted in Matlab. For each experimental condition and within each of the stimulation sites, we used a maximum likelihood estimation to fit a cumulative normal probability distribution. This was achieved through a Generalized Linear Model utilizing the comparison stimulus as the predictor and employing a probit link function. The resulting psychometric functions illustrated the probability of perceiving the comparison stimulus as having a higher vibrating period than the reference stimulus. For each psychometric curve, the Point of Subjective Equality (PSE) was found, representing the vibrating period perceived as faster than the reference in 50% of trials. Additionally, the smallest noticeable difference between stimuli (JND) was calcualated by measuring the gap between the PSE and the vibrating period perceived as faster than the reference in 84% of trials.^10^

For healthy subjects, the statistical analysis was conducted inter-subject, by considering the average value of each subject, with a sample size of N=15 (data from one subject was not properly saved due to a system failure, preventing analysis). Non-blurred (V, T, VT) and blurred conditions (V, VB, VTB) were grouped for each stimulation site. A Repeated Measures ANOVA was conducted, considering condition as the within-subject factor. Post-hoc analysis using the Least Squares Difference LSD method was employed to address multiple comparisons.

Intra-subject statistical analysis was instead conducted for the two amputees. We computed the 95% confidence interval of the PSE and JND distribution from 5000 parametric bootstrap samples with x fixed.^27^^,^^55^ The p-values for the pairwise differences in JNDs were determined by inverting the percentile bootstrap confidence intervals. More in detail the resampling distribution for each JNDs difference was computed and the p-value associated with the largest equi-tailed confidence interval that did not include zero was identified. P-values values were then adjusted via False Discovery Rate to account for the multiple comparisons involved.

#### Statistical analysis: Reaction times

During the 2-AFC task, reaction times were calculated by measuring the interval between the termination of the second stimulus and the moment at which the subject provided their response. First, values above 5 s were discarded as not representative and probable outliers. Then, values equal to more than three standard deviations from the mean were removed as outliers. The statistical analysis of reaction times involved grouping non-blurred (V, T, VT) and blurred conditions (V, VB, VTB) for each stimulation site. For healthy subjects (N=16), inter-subject analysis was conducted by considering the average value of each subject for each stimulation site and experimental condition. A Repeated Measures ANOVA was performed, treating the condition as the within-subject factor, and multiple comparisons were addressed using LSD posthoc. In the case of amputees, intra-subject analysis was carried out. For each analysis, the number of reaction times was the one of the condition with the smallest number of valid (after outliers exclusion) reaction times. Friedman test was employed, followed by LSD post-hoc analysis.

#### Statistical analysis: EEG

In the EEG mental workload analysis, the comparison was carried out across different stimulation sites for the VTB bimodal condition. Again, for each experimental condition, EEG mental workload values equal to more than three standard deviations from the mean were removed as outliers. Data from two healthy subjects were excluded due to connectivity issues. Inter-subject analysis (N=14) was conducted by considering the average value of each subject, for each stimulation site and experimental condition. Repeated Measures ANOVA, with the stimulation site as the within-subject factor, and LSD post-hoc were performed. In amputees, intra-subject analysis was carried out with the repetition of EEG mental workload for each stimulus (N=180) within a single experimental condition. The Wilcoxon signed-rank test was used.
