# Supplementary material for: Amputees but not healthy subjects optimally integrate non-spatially matched visuo-tactile stimuli
Source: iScience. 2024 Dec 25;28(1):111685. doi: 10.1016/j.isci.2024.111685 (PMC11780163; doi:10.1016/j.isci.2024.111685)
Supplement: Document S1. Figures S1–S3 and Tables S1–S8 [file mmc1.pdf]

iScience, Volume *28*

Supplemental information

Amputees but not healthy subjects optimally integrate non-spatially matched visuo-tactile stimuli

Giuseppe Valerio Aurucci, Greta Preatoni, Gaia Risso, and Stanisa Raspopovic

Supplementary materials and results

Fig. S1. Statistical results of the multisensory integration

Fig. S2. Integration probability distribution for non-spatially matched stimuli

Fig. S3. Race model (RM) analysis for reaction times

Table S1. Complete JND results for amputees and healthy subjects

Table S2. JND statistical analysis for healthy subjects

Table S3. JND post-hoc analysis for amputee n.1

Table S4. JND post-hoc analysis for amputee n.2

Table S5. Reaction time statistical analysis for amputee n.1

Table S6. Reaction time statistical analysis for amputee n.2

Table S7. Reaction time statistical analysis for healthy subjects

Table S8. EEG statistical analysis for healthy subjects

Table S9. Patients experimental protocol.

Table S10. Healhy controls experimental protocol.

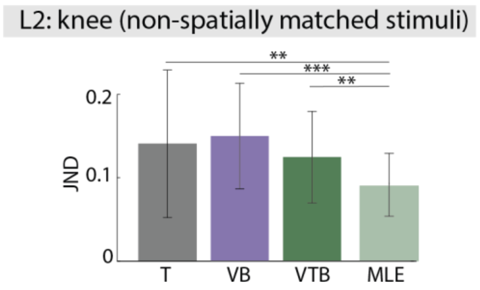

Fig. S1. Statistical results of the multisensory integration. A) Healthy subjects' JND results for L2 (knee). Healthy subjects' JND results for the blurred conditions (T, VB, VTB) in L2 (knee) stimulation site. For JND, the predicted Maximum-Likelihood estimation (MLE) JND is shown as well. Repeated measures ANOVA tests with 'lsd' posthoc were used (N=15). Mean and STD are shown for each bar. * $p < 0.05$, ** $p < 0.01$, *** $p < 0.001$.

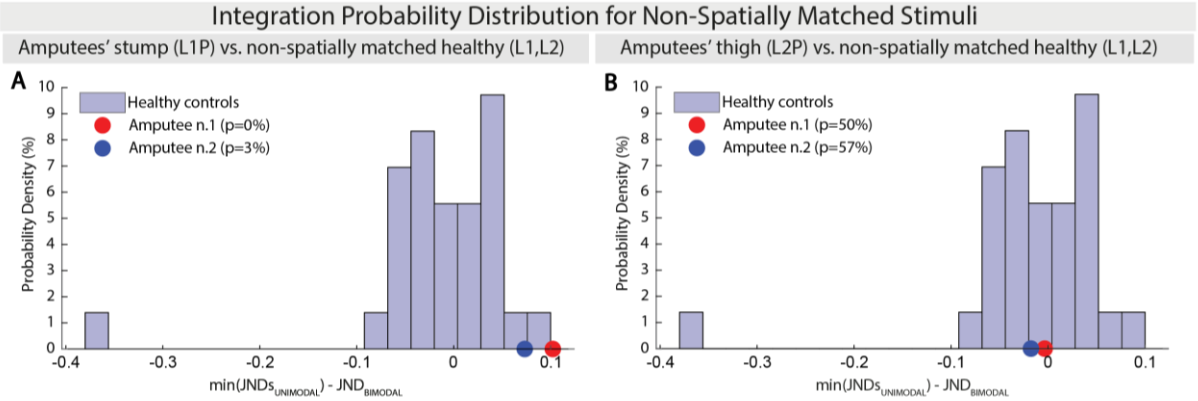

Figure S2. Integration probability distribution for non-spatially matched stimuli. A) Amputees' stump vs. healthy non-spatially matched conditions. The distribution of the relative reduction in bimodal JND, calculated as $(|\mathrm{JND}_{\mathrm{BIMODAL}} - \min(\mathrm{JND}_{\mathrm{UNIMODAL}})\,|$, is shown for healthy controls in non-spatially matched conditions (L1, L2) in light blue. For amputees, the same measure is displayed for the stump (L1P) condition: in red for Amputee 1 and in blue for Amputee 2. The probability that a healthy control exhibits a bimodal JND reduction as strong as Amputee 1 and Amputee 2 is 0% and 3%, respectively. T-tests confirm a significant difference in JND reduction between amputees and healthy controls ($p < 0.001$ for both). B) Amputees' thigh vs. healthy non-spatially matched conditions: The relative reduction in bimodal JND for healthy controls (L1, L2) is again shown in light blue, and for the thigh of the intact leg (L2P) condition in amputees in red (Amputee 1) and blue (Amputee 2). The probability of a healthy control showing a bimodal JND reduction as strong 1 and Amputee 2 are 50% and 57%, respectively. No significant difference is found between amputees and healthy controls for thigh conditions ($p > 0.05$ for both).

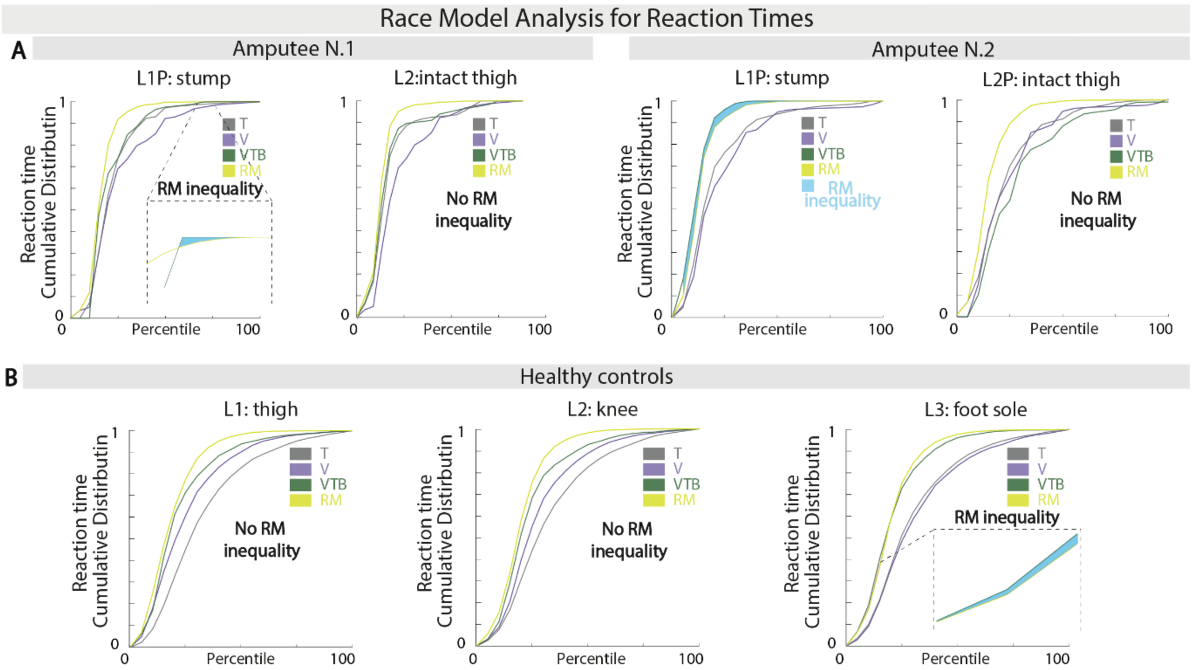

Figure S3. Race model (RM) analysis for reaction times. A) Amputees race model. The RM analysis for amputees examined reaction times for blurred conditions (T, V, VTB) at both stimulation sites (L1P, L2P). Each curve represents the cumulative distribution of reaction rimes for a specific experimental condition and position. RM predictions were calculated at each 5th percentile according to Raab's model [5] as follows: P(RM) = P(T) + P(VB) – P(T) * P(VB). RM inequalities, indicative of effective multisensory integration, were identified when the VTB distribution exceeded the RM prediction at any of the considered bins. Notably, both amputees exhibited RM inequalities exclusively at the stump. B) Healthy subjects race model. The RM analysis for healthy controls evaluated reaction times for blurred conditions (T, V, VTB) across all stimulation sites (L1, L2, L3). Each curve represents the aggregated average across 16 subjects. RM inequalities were observed solely at L3, confirming that multisensory integration occurs only for spatially matched experimental conditions in healthy controls.

Amputee n.1

	T	V	VT	T	VB	VTB	MLE
**L1P**	*0.264 [CI: 0.173, 0.372]*	*0.009 [CI: 0.005, 0.01]*	*0.261 [CI: 0.174, 0.371]*	*0.264 [CI: 0.173, 0.372]*	*0.252 [CI: 0.169, 0.352]*	*0.078 [CI: 0.009, 0.120]*	*0.178 [CI: 0.133, 0.228]*
**L2P**	*0.183 [CI: 0.117, 0.256]*	*0.009 [CI: 0.005, 0.01]*	*0.154 [CI: 0.091, 0.217]*	*0.183 [CI: 0.117, 0.256]*	*0.253 [CI: 0.167, 0.359]*	*0.189 [CI: 0.120, 0.263]*	*0.145 [CI: 0.103, 0.187]*

Amputee n.2

	T	V	VT	T	VB	VTB	MLE
**L1P**	*0.210 [CI: 0.138, 0.289]*	*0.099 [CI: 0.050, 0.147]*	*0.238 [CI: 0.156, 0.334]*	*0.210 [CI: 0.138, 0.289]*	*0.232 [CI: 0.155, 0.324]*	*0.083 [CI: 0.010, 0.128]*	*0.152 [CI: 0.112, 0.194]*
**L2P**	*0.207 [CI: 0.136, 0.287]*	*0.100 [CI: 0.043, 0.148]*	*0.043 [CI: 0.009, 0.092]*	*0.207 [CI: 0.136, 0.287]*	*0.232 [CI: 0.153, 0.320]*	*0.237 [CI: 0.156, 0.334]*	*0.150 [CI: 0.110, 0.192]*

Healthy controls

	T	V	VT	T	VB	VTB	MLE
**L1**	*0.128 +- 0.038*	*0.087 +- 0.04*	*0.096 +- 0.041*	*0.128 +- 0.038*	*0.15 +- 0.063*	*0.131 +- 0.101*	*0.092 +- 0.031*
**L2**	*0.141 +- 0.089*	*0.087 +- 0.04*	*0.074 +- 0.037*	*0.141 +- 0.089*	*0.15 +- 0.063*	*0.125 +- 0.055*	*0.091 +- 0.038*
**L3**	*0.173 +- 0.113*	*0.087 +- 0.04*	*0.092 +- 0.044*	*0.173 +- 0.112*	*0.15 +- 0.063*	*0.09 +- 0.037*	*0.099 +- 0.04*

Table S1. Complete JND results for amputees and healthy subjects. For amputees, each cell contains mean and 95% confidence interval of the bootstrap JND distribution (N=5000). For healthy controls, each cell contains mean $\pm$ STD among N=15 subjects.

Position L1P (LEG) - ANOVA

no-blurred analysis					blurred analysis				
ANOVA					ANOVA				
	SS	df	F	p		SS	df	F	p
conditions	*0.030*	*3*	*11.312*	*1.4 x10^{-5}*	conditions	*0.026*	*3*	*2.491*	*0.07*
error	*0.037*	*42*			error	*0.147*	*42*		

Position L1P (LEG) - posthoc

	T	V	VT	T	VB	VTB	MLE
**T**	*1*	*0.011598, t(3)=2.90, |d|=0.75*	*0.016263361, t(3)=2.73, |d|=0.70*				
**V**	*-*	*1*	*0.441928601, t(3)=0.79, |d|=0.20*				
**VT**	*-*	*-*	*1*				
**T**				*1*	*ns*	*ns*	*ns*
**VB**				*-*	*1*	*ns*	*ns*
**VTB**				*-*	*-*	*1*	*ns*
**MLE**				*-*	*-*	*-*	*1*

Position L2P (KNEE)

no-blurred analysis					blurred analysis				
ANOVA					ANOVA				
	SS	df	F	p		SS	df	F	p
conditions	*0.047*	*3*	*5.607*	*0.0025*	conditions	*0.030*	*3*	*4.65*	*0.007*
error	*0.117*	*42*			error	*0.091*	*42*		

Position L2P (KNEE) - posthoc

	T	V	VT	T	VB	VTB	MLE
**T**	*1*	*0.035, t(3)=2.34, |d|=0.60*	*0.016, t(3)=2.22, |d|=0.57*				
**V**	*-*	*1*	*0.34, t(3)=0.28, |d|=0.07*				
**VT**	*-*	*-*	*1*				
**T**				*1*	*0.719, t(3)=0.37 |d|=0.09*	*0.36, t(3)=0.95 |d|=0.24*	*0.0099,t(3)=2.98 |d|=0.77*
**VB**				*-*	*1*	*0.17, t(3)=1.46 |d|=0.38*	*0.0003,t(3)=4.77 |d|=1.23*
**VTB**				*-*	*-*	*1*	*0.002878745*
**MLE**				*-*	*-*	*-*	*1*

Position L3P (FOOT)

no-blurred analysis					blurred analysis				
ANOVA					ANOVA				
	SS	df	F	p		SS	df	F	p
conditions	*0.095*	*3*	*8.6*	*0.0001*	conditions	*0.071*	*3*	*5,85*	*0.002*
error	*0.155*	*42*			error	*0.171*	*42*		

Position L3P (FOOT) - posthoc

	T	V	VT	T	VB	VTB	MLE
**T**	*1*	*0.011,t(3)=2.94, |d|=0.76*	*0.026,t(3)=2.50,|d|=0.65*				
**V**	*-*	*1*	*0.69,t(3)=0.41,|d|=0.10*				
**VT**	*-*	*-*	*1*				
**T**				*1*	*0.48, t(3)=0.73 |d|=0.19*	*0.026,t(3)=2.49 |d|=0.64*	*0.0064,t(3)=3.20 |d|=0.83*
**VB**				*-*	*1*	*0.0037,t(3)=3.47 |d|=0.90*	*0.0005,t(3)=4.51 |d|=1.16*
**VTB**				*-*	*-*	*1*	*0.56,t(3)=0.58 |d|=0.15*
**MLE**				*-*	*-*	*-*	*1*

Table S2. JND statistical analysis for healthy subjects. For each position, the ANOVA and post-hoc results are shown. For post-hoc, in each cell, the p-value corrected for multiple JND comparisons (Least Square Difference LSD), the t and the Cohen effect size d are shown.

Position L1P

	T	V	VT	T	VB	VTB	MLE
**T**	*1*	*<0.001*	*1*				
**V**	-	*1*	*<0.001*				
**VT**	-	-	*1*				
**T**				*1*	*1*	*<0.001*	*<0.001*
**VB**				-	*1*	*<0.001*	*<0.001*
**VTB**				-	-	*1*	*0.0035*
**MLE**				-	-	-	*1*

Position L2P

	T	V	VT	T	VB	VTB	MLE
**T**	*1*	*<0.001*	*1*				
**V**	-	*1*	*<0.001*				
**VT**	-	-	*1*				
**T**				*1*	*1*	*1*	*<0.001*
**VB**				-	*1*	*0.829*	*<0.001*
**VTB**				-	-	*1*	*1*
**MLE**				-	-	-	*1*

Table S3. JND post-hoc analysis for amputee n.1. In each cell, the p-value corrected for multiple JND comparisons (False Discovery Rate) is shown.

Position L1P

	T	V	VT	T	VB	VTB	MLE
**T**	*1*	*0.034*	*1*				
**V**	-	*1*	*0.176*				
**VT**	-	-	*1*				
**T**				*1*	*1*	*0.012*	*<0.001*
**VB**				-	*1*	*<0.001*	*<0.001*
**VTB**				-	-	*1*	*0.067*
**MLE**				-	-	-	*1*

Position L2P

	T	V	VT	T	VB	VTB	MLE
**T**	*1*	*0.0514*	*0.002*				
**V**	-	*1*	*0.456*				
**VT**	-	-	*1*				
**T**				*1*	*1*	*1*	*<0.001*
**VB**				-	*1*	*1*	*<0.001*
**VTB**				-	-	*1*	*0.288*
**MLE**				-	-	-	*1*

Table S4. JND post-hoc analysis for amputee n.2. In each cell, the p-value corrected for multiple JND comparisons (False Discovery Rate) is shown.

Position L1P - blurred conditions
Friedman table

	SS	df	Chi-sq	p
**conditions**	*9.377*	*2*	*9.38*	*0.009*
**error**	*144.623*	*152*		

posthoc

	T	VB	VTB
**T**		*p=0.375,t(2)=1.19,|d|=0.20*	*p=0.036,t(2)=0.91,|d|=0.14*
**VB**			*p=0.029,t(2)=2.06,|d|=0.33*
**VTB**			

Position L2P - blurred conditions
ANOVA

	SS	df	Chi-sq	P
**conditions**	*9.117*	*2*	*9.12*	*0.011*
**error**	*144.883*	*152*		

Posthoc

	T	VB	VTB
**T**		*p=0.0296,t(2)=2.19,|d|=0.34*	*p=0.468,t(2)=0.40,|d|=0.06*
**VB**			*p=0.004,t(2)=2.63,|d|=0.38*
**VTB**			

Table S5. Reaction time statistical analysis for amputee n.1. For each position, the ANOVA and post-hoc results are shown. For post-hoc, in each cell, the p-value corrected for multiple JND comparisons (Least Square Difference LSD), the t and the Cohen effect size d are shown.

Position L1P - blurred conditions
Friedman table

	SS	df	Chi-sq	p
**conditions**	*26.831*	*2*	*26.83*	*< 0.001*
**error**	*127.169*	*152*		

posthoc

	T	VB	VTB
**T**		*p=0.68,t(2)=0.76,|d|=0.13*	*P<0.001,t(2)=4.39,|d|=0.71*
**VB**			*P<0.001,t(2)=5.45,|d|=0.80*
**VTB**			

Position L2P - blurred conditions
ANOVA

	SS	df	Chi-sq	P
**conditions**	*0.935*	*2*	*0.94*	*0.62*
**error**	*153.065*	*152*		

Table S6. Reaction time statistical analysis for amputee n.2. For each position, the ANOVA and post-hoc results are shown. For post-hoc, in each cell, the p-value corrected for multiple JND comparisons (Least Square Difference LSD), the t and the Cohen effect size d are shown.

Position L1 - blurred conditions

ANOVA

	SS	df	F	p
**conditions**	*0.779*	*2*	*5.88*	*0.007*
**error**	*1.984*	*30*		

posthoc

	T	VB	VTB
**T**		*p=0.048,t(2)=2.16,|d|=0.54*	*p=0.159,t(2)=1.48,|d|=0.37*
**VB**			*p=0.011,t(2)=2.90,|d|=0.72*
**VTB**			

Position L2 - blurred conditions

ANOVA

	SS	df	F	p
**conditions**	*0.61*	*2*	*2.63*	*0.088*
**error**	*3.52*	*30*		

Position L3 - blurred conditions

ANOVA

	SS	df	F	p
**conditions**	*1.341*	*2*	*6.88*	*0.003*
**error**	*2.92*	*30*		

posthoc

	T	VB	VTB
**T**		*p=0.565,t(2)=0.59,|d|=0.87*	*p=0.003,t(2)=3.47,|d|=0.87*
**VB**			*p=0.004,t(2)=3.39,|d|=0.85*
**VTB**			

Table S7. Reaction time statistical analysis for healthy subjects. For each position, the ANOVA and post-hoc results are shown. For post-hoc, in each cell, the p-value corrected for multiple JND comparisons (Least Square Difference LSD), the t and the Cohen effect size d are shown.

Condition VTB

ANOVA

	SS	df	F	p
**positions**	*2.342*	*2*	*4.63*	*0.019*
**error**	*6.589*	*26*		

posthoc

	LEG	KNEE	FOOT
**LEG**		*p=0.18,t(2)=1.41,|d|=0.36*	*p=0.021,t(2)=2.63,|d|=0.68*
**KNEE**			*p=0.026,t(2)=2.52,|d|=0.65*
**FOOT**			

Table S8. EEG statistical analysis for healthy subjects. ANOVA and post-hoc results are shown for the bimodal blurred conditions across the tree stimulation sites. For post-hoc, in each cell, the p-value corrected for multiple JND comparisons (Least Square Difference LSD), the t and the Cohen effect size d are shown.

Amputee 1	*L1P-VT*	*VB*	*L1P-VTB*	*V*	*L1P-T*	*L2P-VT*	*L2P-T*	*L2P-VTB*
Amputee 2	*VB*	*V*	*L1P-VTB*	*L1P-T*	*L1P-VT*	*L2P-VT*	*L2P-VTB*	*L2P-T*

Table S9. Patients experimental protocol.

S01	*V*	*L3-T*	*L3-VTB*	*VB*	*L3-VT*	*L2-VT*	*L2-T*	*L2-VTB*	*L1-T*	*L1-VT*	*L1-VTB*
S02	*L2-T*	*L2-VTB*	*VB*	*L2-VT*	*V*	*L3-VT*	*L3-T*	*L3-VTB*	*L1-VTB*	*L1-T*	*L1-VT*
S03	*L1-VT*	*VB*	*L1-VTB*	*V*	*L1-T*	*L3-T*	*L3-VT*	*L3-VTB*	*L2-T*	*L2-VTB*	*L2-VT*
S04	*V*	*L3-T*	*L3-VT*	*VB*	*L3-VTB*	*L1-T*	*L1-VTB*	*L1-VT*	*L2-T*	*L2-VTB*	*L2-VT*
S05	*L3-VTB*	*V*	*L3-T*	*L3-VT*	*VB*	*L1-VT*	*L1-VTB*	*L1-T*	*L2-VT*	*L2-VTB*	*L2-T*
S06	*L1-T*	*L1-VT*	*L1-VTB*	*VB*	*V*	*L2-T*	*L2-VTB*	*L2-VT*	*L3-T*	*L3-VTB*	*L3-VT*
S07	*L2-T*	*L2-VT*	*VB*	*L2-VTB*	*V*	*L3-VTB*	*L3-T*	*L3-VT*	*L1-VT*	*L1-VTB*	*L1-T*
S08	*VB*	*L3-T*	*L3-VTB*	*V*	*L3-VT*	*L2-VTB*	*L2-T*	*L2-VT*	*L1-VT*	*L1-T*	*L1-VTB*
S09	*L2-T*	*VB*	*L2-VTB*	*V*	*L2-VT*	*L3-T*	*L3-VTB*	*L3-VT*	*L1-VT*	*L1-T*	*L1-VTB*
S10	*V*	*L3-T*	*VB*	*L3-VTB*	*L3-VT*	*L1-VT*	*L1-VTB*	*L1-T*	*L2-T*	*L2-VTB*	*L2-VT*
S11	*L2-VTB*	*V*	*VB*	*L2-VT*	*L2-T*	*L1-VT*	*L1-VTB*	*L1-T*	*L3-T*	*L3-VT*	*L3-VTB*
S12	*VB*	*L2-VTB*	*V*	*L2-T*	*L2-VT*	*L1-T*	*L1-VTB*	*L1-VT*	*L3-T*	*L3-VT*	*L3-VTB*
S13	*VB*	*L1-VT*	*L1-T*	*V*	*L1-VTB*	*L3-VT*	*L3-T*	*L3-VTB*	*L2-VT*	*L2-VTB*	*L2-T*
S14	*L1-VTB*	*L1-VT*	*VB*	*V*	*L1-T*	*L3-VT*	*L3-VTB*	*L3-T*	*L2-VT*	*L2-VTB*	*L2-T*
S15	*L3-T*	*VB*	*L3-VT*	*L3-VTB*	*V*	*L2-VT*	*L2-VTB*	*L2-T*	*L1-T*	*L1-VTB*	*L1-VT*
S16	*L1-T*	*VB*	*L1-VT*	*L1-VTB*	*V*	*L3-VT*	*L3-VTB*	*L3-T*	*L2-VT*	*L2-T*	*L2-VTB*

Table S10. Healthy controls experimental protocol.

[1] D. H. Raab, ‘Division of Psychology: Statistical Facilitation of Simple Reaction Times’, *Transactions of the New York Academy of Sciences*, vol. 24, no. 5 Series II, pp. 574–590, 1962, doi: 10.1111/j.2164-0947.1962.tb01433.x.
